# Supplementary material for: Quantitative In-Depth Analysis of the Mouse Mast Cell Transcriptome Reveals Organ-Specific Mast Cell Heterogeneity
Source: Cells. 2020 Jan 14;9(1):211. doi: 10.3390/cells9010211 (PMC7016716; doi:10.3390/cells9010211)
Supplement: Supplementary file 1 [file cells-09-00211-s001.pdf]

cells

MDPI

Suppl. Table 1. Transcript levels for granule proteases in mouse peritoneal MCs (from BALB/c mice). The number of normalized reads for each of the different genes is given in actual numbers (obtained from Ampliseq analyses). The MC sample in this analysis was one of 16 samples on the chip. The remaining RNA samples represented the brain, tongue, liver, heart, pancreas, duodenum, colon, kidney, spleen and uterus. In the comments the expression levels of the various genes in the MC transcriptome are related in expression to the other 10 tissues in this Ampliseq analysis.

Gene	Reads	Comments
Mcpt5 (Cma1)	45221	MC-specific
Mcpt4	33040	MC-specific
Mcpt6 (Tpsb2)	67773	MC-specific
Cpa3	45604	MC- and basophil-specific
Mcpt7 (Tpsab1)	386	MC-specific
CtsG	1111	Expressed by neutrophils and also at lower levels by MCs
Tpsg1 (tryptase gamma)	123	More highly expressed in MCs compared to other tissues colon closest (28 reads)
Mcpt11 (Prss34)	25	Very highly expressed in MCs compared to other tissues
Mcpt8	38	Basophil-specific
Mcpt2	1	Expressed primarily by mucosal MCs
Mcpt1	0.3	Mucosal MC-specific
CtsC (DPP)	51	Expressed in several hematopoietic cells, an activator of hematopoietic serine proteases
GzmB	581	Expressed primarily by cytotoxic T cells
GzmA	3	Expressed primarily by cytotoxic T cells and NK cells
GzmK	10	Expressed primarily by cytotoxic T cells and NK cells
GzmM	1	Expressed primarily by NK cells
GzmN	1	Expressed primarily by cytotoxic T cells
GzmC, D, E, F, G	0	Expressed primarily by cytotoxic T cells
CtsE	1248	Cathepsin E; involved in protein degradation; High also in duodenum (1400 reads)
Plau	661	Urokinase; High in MCs and kidney
Tpp1	588	Tripeptidyl peptidase 1; High in MCs and Kidney
Adamts9	370	Very high compared to other tissues, e.g. uterus (85 reads)
Usp11	198	Ubiquitin carboxyl-terminal hydrolase; High compared to other tissues, e.g., brain (132 reads)

Suppl. Table 2. Transcript levels for protease inhibitors in mouse peritoneal MCs (from BALB/c mice). The number of normalized reads for each of the different genes is given in actual numbers (obtained from Ampliseq analyses).

Gene name	Reads	Comments
Serpin b1a	1037	Much higher in MCs than other tissues closest colon (241 reads)
Serpin b6a	127	Similar to other tissues; probably higher in MQ

Lxn	185	inhibitor of metallo exo-peptidases); Brain (70 reads)
Cst7 (Cystatin F)	181	Much higher in MCs than in other tissues

Suppl. Table 3. Transcript levels for receptors (Ig receptors and related) in mouse peritoneal MCs (from BALB/c mice). The number of normalized reads for each of the different genes is given in actual numbers (obtained from Ampliseq analyses).

Gene name	Reads	Comments
FcεRI alpha	345	More than 300 times higher in MCs compared to other tissues
FcεRI beta (Ms4a2)	1297	More than 600 times higher in MCs compared to other tissues, spleen 1.9
FcεRI gamma	2913	Very high in MCs compared to other tissues; spleen closest (206 reads)
FcgRIII alpha	752	Very high in MCs compared to other tissues; spleen closest (57 reads)
FcgRIIb	91	Very high in MCs compared to other tissues; spleen closest (19 reads)
FcmuR (FcmR)	23	Possibly from a minor B cell contamination
FcRLA	11	Possibly from a minor B cell contamination
FcRLB	0.2	Possibly from a minor B cell contamination
FcRL1	9	Possibly from a minor B cell contamination
FcRL5	3	Possibly from a minor B cell contamination
FcgRIV	5	Possibly from a minor B cell contamination
FcgRI	2	Possibly from a minor MQ contamination
MilR1	283	Mast Cell Immunoglobulin Like Receptor 1, Allergin-1; 100 times higher in MCs than in any of the other tissues

Suppl. Table 4. Transcript levels for cytokine and chemokine receptors in mouse peritoneal MCs (from BALB/c mice). The number of normalized reads for each of the different genes is given in actual numbers (obtained from Ampliseq analyses).

Gene name	Reads	Comments
Gfra2	2016	Member of GDNF receptor family; binds GDNF and neurturin (NTN), a glycosylphosphatidylinositol (GPI) linked cell surface neurotrophin receptor; Very low in most other tissues except brain (62 reads)
c-kit	1198	Stem cell factor (SCF) receptor. Very high in MCs (100 x) compared to other tissues; spleen closest (12 reads)
IL-3 Ra	159	Very low in most other tissues except uterus (16 reads)
Csf2rb	415	common beta chain IL-3, 5 and GM-CSF; Very high in MCs compared to other tissues; uterus closest (42 reads)
Il1rl1 (ST2)	1693	IL-33 receptor; Very high in MCs compared to other tissues; spleen closest (6 reads)
IL12rb2	42	IL-12 receptor beta 2; More than 10 times higher in MCs than in other tissues

Ifnar2	453	Interferon alpha/beta receptor beta; High in MCs compared to other tissues; uterus closest (120 reads)
Ifnar1	90	Interferon alpha/beta receptor alpha; similar levels in other tissues
Ifngr1	228	Interferon gamma receptor alpha; similar levels in other tissues
Ifngr2	115	Interferon gamma receptor beta; similar levels in other tissues

Suppl. Table 5. Transcript levels for MAS GPR receptors in mouse peritoneal MCs (from BALB/c mice). The number of normalized reads for each of the different genes is given in actual numbers (obtained from Ampliseq analyses).

Gene name	Reads	Comments
Mrgprb2	899	MAS-related GPR, member B2; 800 times higher in MCs compared to other tissues
Mrgprb1	366	MAS-related GPR, member B1; 30 times higher in MCs compared to other tissues
Mrgpra4 s	155	MAS-related GPR, member A4; More than 200 times in MCs higher compared to other tissue
Mrgprb8	19	MAS-related GPR, member B8; More than 40 times higher in MCs compared to other tissues
Mrgprx2	16	MAS-related GPR, member x2; More than 5 times higher in MCs compared to other tissues
Mrgprx1 es	13	MAS-related GPR, member x1; More than 13 times higher in MCs compared to other tissues
Mrgprb4	11	MAS-related GPR, member B4; More than 30 times higher in MCs compared to other tissues

Suppl. Table 6. Transcript levels for endothelin-, ATP-, histamine- and adrenergic receptors in mouse peritoneal MCs (from BALB/c mice). The number of normalized reads for each of the different genes is given in actual numbers (obtained from Ampliseq analyses).

Gene name	Reads	Comments
Ednra	892	Endothelin A receptor; Very high in MCs compared to other tissues; heart closest (66 reads)
Adrb2	631	beta-2 adrenergic receptor-associated with asthma and obesity; Very high in MCs compared to other tissues; spleen closest (42 reads)
P2rx7	571	P2X purino receptor 7 binds and respond to ATP; role in MC degranulation and sensing of apoptotic cells; Very high in MCs compared to other tissues; spleen closest (35 reads)
P2rx1	327	P2X purino receptor 1; binds and respond to ATP; Very high in MCs compared to other tissues; spleen closest (9 reads)

Hrh4	25	Histamine receptor 4; High in MCs compared to all other tissues; spleen closest (0.3 reads)

Suppl. Table 7. Transcript levels for receptors (others) in mouse peritoneal MCs (from BALB/c mice). The number of normalized reads for each of the different genes is given in actual numbers (obtained from Ampliseq analyses).

Gene name	Reads	Comments
Fxyd5	1850	Dysadherin; Much higher in MCs than in in other tissues; spleen closest (104 reads)
Lat2	1588	Linker of activation of T cell family members; MC activating function; High in MCs compared to other tissues; spleen closest (142 reads)
Adgrl1	297	Latrophilin adhesion-GPCR family of receptors; Very high in MCs compared to other tissues except brain (476 reads)
Ramp1	308	Receptor activity modifying protein 1; role in transport of the calcitonin gene-related peptide (CGRP) receptor to the membrane; Very high in MCs compared to other tissues except brain (224 reads)
Amhr2	39	Receptor for anti-Mullerian hormone; More than 7 x higher in MCs than in any other tissue
Slc6a2	191	Sodium and chloride dependent betaine transporter; Much higher in MCs than in other tissues except Liver (48 reads)
Gpr162	238	G coupled receptor 162; Mainly expressed in MCs and brain (brain: 198 reads)
Tarm1	156	T-cell-interacting; activating receptor on myeloid cells protein 1; Very in MCs high compared to all other tissues, more than 100 times

Suppl. Table 8. Transcript levels for Cluster of differentiation (CD) receptors in mouse peritoneal MCs (from BALB/c mice). The number of normalized reads for each of the different genes is given in actual numbers (obtained from Ampliseq analyses).

Gene name	Reads	Comments
CD34	332	Higher than in most other tissues uterus closest (180 reads)
CD81	1475	High also in other tissues, kidney closest (660 reads)
CD55	439	Decay accelerating factor DAF; High in MCs compared to other tissues spleen closest (80 reads).
CD200R3	275	Cell surface glycoprotein; 100-1000 times higher in MCs than in other tissues; MC-activating
CD300a	184	Inhibitory receptor (four cytoplasmic ITAMS) high also in spleen (191 reads)
CD274	200	PD-L1; 5–100 fold higher than other tissues spleen closest (34 reads)

CD276	163	B7-H3; Much higher in MCs than in other tissues; duodenum closest (11 reads)
CD40lg	0.1	CD40 ligand; Almost absent
CD40	3.8	CD40; Almost absent

Suppl. Table 9. Transcript levels for TLRs, olfactory- and vomeronasal receptors in mouse peritoneal MCs (from BALB/c mice). The number of normalized reads for each of the different genes is given in actual numbers (obtained from Ampliseq analyses).

Gene name	Reads	Comments
Tlr4	61	Slightly higher in MCs than in other tissues; uterus closest (53 reads)
Tlr13	13	Much higher in spleen than in MCs (52 reads); most likely expressed in MQ
Olfr	2-18	40–50 different receptors
Vmn1r family members	2-15	Vomeronasal receptors-pheromone binding; low level expression; among these 11 tissues only detected in the MC; same range as the olfactory receptors

Suppl. Table 10. Transcript levels for cell adhesion molecules in mouse peritoneal MCs (from BALB/c mice). The number of normalized reads for each of the different genes is given in actual numbers (obtained from Ampliseq analyses).

Gene name	Reads	Comments
Adgre5	2838	CD97, member of the GPCR family of adhesion receptors; High among hematopoietic cells primarily myeloid spleen closest (184 reads)
Itga4	404	Integrin alpha 4; High in MCs compared to other tissues (spleen:82 reads)
Itgb1	444	Integrin beta 1; High also in many other tissues
Itgb2	388	Integrin beta 2; Higher in MCs compared to other tissues spleen (259 reads) probably high in MQ
Itga2b	266	Integrin alpha 2b; 10-100 times higher in MCs compared to other tissues
Itga9	217	Integrin alpha 9; 3-100 x higher in MCs compared to other tissues; heart closest (64 reads)
Itga5	135	Integrin alpha 5; High also in other tissues
Itgam	113	Integrin alpha m; Higher in MQ; high also in many other tissues
Itgb	211	Integrin beta 7; 4-100 x higher in MCs compared to other tissues; spleen closest (42 reads)
Itga2	137	Integrin alpha 2; High in MCs compared to other tissues; duodenum closest (22 reads)
Selplg1	369	P selectin ligand CD162; High in MCs compared to other tissues except spleen (82 reads)
Ncam1	145	Neural adhesion molecule 1; also named CD56; High in MCs but lower than in brain (279 reads)

Muc13	147	Mucin 13; High in MCs but lower than in duodenum and colon (520 and 1178 reads, respectively)
Spn	134	CD43 Sialophorin; High in MCs compared to other tissues; spleen closest (17 reads)

Suppl. Table 11. Transcript levels for genes involved in synthesis of proteoglycans, histamine, serotonin, lipid mediators and steroid hormones in mouse peritoneal MCs (from BALB/c mice). The number of normalized reads for each of the different genes is given in actual numbers (obtained from Ampliseq analyses).

Gene name	Reads	Comments
Srgn	8905	Serglycin; Expressed by several hematopoietic cell types
Ndst 2	1112	N-deacetylase/N-sulfotransferase 2, Very high in MCs compared to other tissues; spleen closest (22 reads)
Ndst 1	38	
Ndst 3	0	
Ext1	254	Exostosin-1; an endoplasmic reticulum-resident type II transmembrane glycosyltransferase involved in the chain elongation step of heparan sulfate biosynthesis; 5-10 x higher in MCs than in other tissues
Chst15	448	N-acetylgalactosamine 4-sulfate 6-O-sulfotransferase; High in MCs compared to other tissues
Ids	657	Iduronate-2-sulphatase; High also in brain (579 reads)
Gfpt1	398	Glucosamine-fructose-6-phosphate aminotransferase isomerizing 1; High in MCs compared to most other except duodenum (1600 reads) and tongue (552 reads)
St6galnac3	290	N-acetylgalactosaminide Alpha-2,6-Sialyltransferase 3; High (10-100 x) in MCs compared to other tissues
Chst11	216	Carbohydrate sulfotransferase 11; High in MCs compared to other tissues (brain: 48 reads)
Sgsh	215	N-sulfoglucosamine sulfohydrolase; 7-100 fold higher in MCs than in other tissues
Hs6st2	152	Heparan sulfate 6-O-sulfotransferase 2; High in MCs compared to other tissues (brain: 47 reads)
Slc35d1	187	resides in the ER; transports UDP- glucuronic acid and UDP-N-acetylgalactosamine; 4-100 x higher in MCs than in other tissues (liver 52 reads; tongue 50 reads)
Hdc	936	Histidine decarboxylase. High compared to other tissues kidney closest (28 reads)
Slc18a2	861	Mono amine transporter (e.g. histamine); Very high in MCs compared to other tissues brain closest (4 reads) or 200 times

Tph1	550	Tryptophan hydroxylase; forms serotonin; Very high in MCs compared to other tissues duodenum closest (5 reads) 100 times
Slc6a4	1468	Serotonin transporter; 50 x higher in MCs compared to other tissues
PLA2g7	1116	Phospholipase A2; Very high in MCs compared to other tissues brain closest (276 reads)
Alox5	486	5-Lipoxygenase; Very high in MCs compared to other tissues uterus closest (5 reads) or 100 times.
Hpgds	450	Hematopoietic prostaglandin D synthase; Very high in MCs compared to other tissues, spleen closest (27 reads)
Lpcat2	177	Lysophosphatidylcholine acyltransferase 2; role in PAF synthesis; Much higher in MCs than in other tissues; duodenum closest (28 reads)
Dapp1	389	Dual adapter for phosphotyrosine and 3-phosphotyrosine and 3-phosphoinositide; Very high in MCs compared to other tissues, spleen closest (15 reads)
Plcb2	211	1-Phosphatidylinositol-4,5-bisphosphate phosphodiesterase beta-2 phospholipase C β2; 14-100 x higher in MCs than in other tissues; spleen closest (15 reads)
Slc 45a3	673	Solute carrier protein; High in MCs compared to other tissues
Slc7a5	402	Large neutral amino acid transporter light chain; High in MCs compared to other tissues (duodenum 55 reads; colon 121 reads)
Maob	359	Mono amine oxidase B; Very high in MCs compared to other tissues except liver (190 reads)
Osbpl8	738	Lipid receptor, (oxysterol); High in MCs compared to other tissues kidney closest (92 reads)
Abcb1b	336	ATP-dependent translocase ABCB1; translocates phospholipids between the outer and inner side of membranes; Very high (10-100 x) in MCs compared to other tissues
Acer	214	Alcaline ceramidase 3; 4-100 x higher in MCs than in other tissues; spleen closest (47 reads)
Esyt3	134	Extended synaptogamin-3; lipid transport; Much higher in MCs than other tissues except tongue (68 reads)
Cyp11a1	1217	Cholesterol side-chain cleavage enzyme P450scc; involved in steroid hormone generation (first step from cholesterol); 300 x higher in MCs than in other tissues.
Gyg	389	Glycogenin enzyme forming glycogen; High in MCs compared to other tissues except heart (214 reads)

Suppl. Table 12. Transcript levels for cytokines and chemokines in mouse peritoneal MCs (from BALB/c mice). The number of normalized reads for each of the different genes is given in actual numbers (obtained from Ampliseq analyses).

Gene name	Reads	Comments
Ccl2	397	MCP1; monocyte chemoattractant protein 1; Very high in MCs compared to other tissues; uterus closest (7 reads)
Ccl6	188	only expressed in mouse; expressed by MQ and neutrophils; High in several other tissues
Fbrs	355	fibrosin 1; lymphokine inducing fibroblast proliferation; High in MCs compared to other tissues; tongue closest (90 reads)
Tgfb1	550	TGF beta 1; High in MCs compared to other tissues; spleen closest (109 reads)
Nrros	180	TGF beta activator LRRC33; Much higher (10-100 x) in MCs than all other tissues
IL-4	13	Much higher in MCs than in other tissues
IL-5	0	
IL-13	4	Much higher in MCs than in other tissues
IL15	8	
IL-18	18	
IL-1alpha	6	
IL-6	12	
IL-7	0.1	
IL-10	0	
IL-25	0	
IL-11	0	
IL-12b	9	Higher in MCs than in other tissues
IL-33	0	
IL-31	0	
Hgf	59	Hepatocyte growth factor; Higher in MCs than in other tissues liver closest (22 reads)
Osm	38	Oncostatin; Higher in MCs than in other tissues spleen closest (2 reads)

Suppl. Table 13. Transcript levels for transcription factors in mouse peritoneal MCs (from BALB/c mice). The number of normalized reads for each of the different genes is given in actual numbers (obtained from Ampliseq analyses).

Gene name	Reads	Comments
GATA-2	2272	Much higher in MCs than in other tissues; kidney closest (45 reads)
GATA 1	74	Higher in MCs than in other tissues; spleen closest (37 reads)
GATA 3	27	Higher in MCs than in other tissues; kidney closest (25 reads)
MITF	370	Much higher in MCs than in other tissues; heart closest (34 reads)

Myb	2490	Member of SANT/Myb family of transcription factors; Very high in MCs compared to other tissues; colon closest (105 reads)
Zeb2	1018	Zink finger E box homeobox 2; Higher in MCs than in other tissues; uterus closest (290 reads)
CREB 3l1	992	Stress-induced transcription factor; Very high in MCs compared to other tissues; Duodenum also high (300 reads)
Fam129 b	663	Negative regulator of apoptosis; High in MCs compared to other tissues except tongue with 355 reads
Runx1	638	transcription factor CBF-alpha family hematopoietic development; High in MCs; spleen closest (61 reads)
Runx3	409	May be regulated by MITF; Very high in MCs compared to other tissues; spleen closest (2 reads)
TOX2	568	Very high in MCs compared to most other tissues except brain (75 reads)
Fli1	473	Also known as transcription factor ERGB; High in MCs compared to other tissues; uterus closest (117 reads)
CREBbp	426	CREB binding protein transcription factor; High in MCs compared to other tissues; tongue closest (115 reads)
Pnn	423	Pinin transcription factor E box binding CAGGTG; High in MCs compared to other tissues; spleen closest (60 reads)
Ldb1	423	LIM domain binding transcription factor; High in MCs compared to other tissues; spleen closest (144 reads)
Cbfa2t3	418	transcription regulator; interacts with HDACs and Runx; Very high in MCs compared to other tissues; brain closest (35 reads)
Crtc3	369	CREB-regulated transcription coactivator 3; Very high in MCs compared to other tissues; uterus closest (40 reads)
Atf7ip	357	Activating transcription factor 7-interacting protein 1; 3-4 x higher in MCs than in other tissues
Kmt2c	346	Lysine N-methyltransferase 2C, myeloid/lymphoid; 10 x higher in MCs compared to other tissues
Phf21a	343	PHD finger protein 21A; silences neuronal genes; 6-10 x higher in MCs than in other tissues
Meis2	300	Homeobox protein; 6-10 x higher in MCs than in other tissues
Cited4	277	Cbp/P300 Interacting Transactivator With Glu/Asp Rich Carboxy-Terminal Domain 4; 5-100 x higher in MCs than in other tissues
Ankrd12	269	Ankyrin repeat domain-containing protein 12; May recruit HDACs to the p160 coactivators/nuclear receptor complex to inhibit ligand-dependent transactivation; 5-10 times higher in MCs than in other tissues
Tal1	246	Basic helix-loop-helix transcription factor; 15-100 x higher in MCs than in other tissues
Samsn1	187	Sam domain containing; negative regulator of B cell activation; Much higher in MCs than any other tissue except spleen (49 reads)

Suppl. Table 14. Transcript levels for genes coding for RNA-binding proteins in mouse peritoneal MCs (from BALB/c mice). The number of normalized reads for each of the different genes is given in actual numbers (obtained from Ampliseq analyses).

Gene name	Reads	Comments
Luc7l2	558	RNA binding; High in MCs compared to other tissues (spleen: 97 reads)
Prpf38b	540	Splicing factor; High in MCs compared to other tissues (spleen: 83 reads)
Rbm5	538	RNA binding; regulates differential splicing; High in MCs compared to other tissues (spleen: 83 reads)
Celf2	398	RNA binding; High in MCs compared to other tissues except brain (207 reads)
Lyl1	363	Hematopoietic; possible interaction with Nf꩜B; Very high in MCs compared to other tissues (spleen: 55 reads)
Pnisr	345	Splicing factor; arginine/serine-rich 18; High in MCs compared to other tissues (spleen: 140 reads)
Ptbp3	345	Polypyrimidine tract-binding protein 3; High in MCs compared to other tissues (tongue: 141 reads)
Rsrc1	203	Ser/Arg related protein 53 plays a role in splicing; Higher in MCs than in most other tissues; brain closest (165 reads)

Suppl. Table 15. Transcript levels for genes involved in cell division in mouse peritoneal MCs (from BALB/c mice). The number of normalized reads for each of the different genes is given in actual numbers (obtained from Ampliseq analyses).

Gene name	Reads	Comments
Tbc1d8	682	Role in cell cycle
Kmt2e	484	PHD zinc finger; cell cycle related function; High in MCs compared to other tissues; uterus closest (105 reads)
Smc5	340	Structural maintenance of chromosomes protein 5; High in MCs compared to other tissues (spleen: 35 reads)
Msh5	285	MutS protein homolog 5; involved in mismatch repair and recombination; 15-100 x in MCs higher than in other tissues
Dock10	389	Dedicator of cytokinesis 10; Very high in MCs compared to other tissues except spleen (90 reads)
Dock2	250	Dedicator of cytokinesis 2; Higher (6-100 fold) in MCs compared to other tissues except spleen (42 reads)
Dock11	265	Dedicator of cytokinesis 11 (also known as Zizimin2); Much higher in MCs than in other tissues except spleen (54 reads)
Cdk6	236	Cell division protein kinase 6; Much higher in MCs (12-100 x) than in other tissues
Anapc15	213	Anaphase promoting complex subunit 15; 5-100 x higher in MCs than in other tissues (spleen: 38 reads)

Suppl. Table 16. Transcript levels for genes involved in cell signaling in mouse peritoneal MCs (from BALB/c mice). The number of normalized reads for each of the different genes is given in actual numbers (obtained from Ampliseq analyses).

Gene name	Reads	Comments
Prkcb	1001	PKC beta; Very high in MCs compared to most other tissues, except brain (344 reads)
Rac2	796	Rac family signaling, botulinium substrate; Very high in MCs compared to other tissues; spleen closest (133 reads)
Pik3r6	767	PI3K gamma sub; Very high compared to other tissues; uterus closest (30 reads)
Rassf4	707	Ras associated; controls calcium flux; High in MCs compared to other tissues; brain closest (35 reads)
Arhgap18	697	Rho activating cell signaling; High in MCs compared to other tissues; kidney closest (80 reads)
Plek	891	Plekstrin; binds phosphatidyl inositol lipids; High in MCs compared to other tissues; spleen closest (149 reads)
Inpp5d	664	Src homology domain phosphatase; High in MCs compared to other tissues (spleen: 37 reads)
Inpp4b	558	Inositol polyphosphate-4-phosphatase type II; involved in PI signaling; High in MCs compared to other tissues (spleen: 103 reads)
Rapgef2	624	Rap guanine nucleotide exchange factor 2; link between cell surface receptors and RAS activation; High in MCs compared to other tissues; spleen closest (149 reads)
Tiam1	597	Rho binding regulates extracellular signals to cytoskeletal activities; High in MCs compared to other tissues (tongue: 157 reads)
Sla	571	Src like adaptor regulates receptor signaling; High in MCs compared to other tissues (spleen: 74 reads)
Fam198b	566	Kinase family member of unknown function; High in MCs compared to other tissues (uterus: 125 reads)
Lcp2	514	Lymphocyte cytosolic protein 2; role in signal transduction; High in MCs compared to other tissues (spleen: 79 reads)
Rgs18	509	Regulator of G protein signaling; Very high in MCs compared to other tissues (spleen: 21 reads)
Gnaz	462	G protein member that may have functions regulating lymphatic ion balance; Very high in MCs compared to other tissues except brain (110 reads)
Ptpn2	437	Tyrosine-protein phosphatase non-receptor type 2; High in MCs compared to other tissues (spleen: 52 reads)
Gpsm3	435	G protein signaling modulator 3; Very high in MCs compared to other tissues except spleen (135 reads)

Ptpn6	401	Tyrosine-protein phosphatase non-receptor type 6; Very high in MCs compared to other tissues except spleen (131 reads)
Ptpn7	393	Tyrosine-protein phosphatase non-receptor type 6; Very high in MCs compared to other tissues except spleen (37 reads)
Dnah8	250	Dynein heavy chain 8; axonemal; microtubule based movement flagella; 8-200 x higher in MCs than in other tissues
Gng2	377	Guanine nucleotide-binding protein subunit gamma-2; High in MCs compared to other tissues except brain (210 reads)
Kidins220	371	Kinase D-interacting substrate of 220 kDa; High in MCs compared to other tissues (kidney: 148 reads)
Prkcd	350	PKC delta; High in MCs compared to other tissues; duodenum closest (70 reads)
Inppk5	337	Inositol Polyphosphate- 5-Phosphatase K; High in MCs compared to other tissues; uterus closest (78 reads)
Gcsam	311	Germinal center-associated signaling and motility protein; Very high in MCs compared to other tissues (spleen: 9 reads)
Rab44	302	Ras oncogene member 44; Very high (≥ 100 x) in MCs compared to other tissues
Rgs13	289	Regulator of G-protein signaling 13; suppresses IgE-mediated allergic responses; Very high (40-100 x) in MCs compared to other tissues
Arhgef2	288	Rho guanine nucleotide exchange factor 2; 5-100 x higher in MCs than in other tissues.
Mlph	285	Melanophilin member of the exophilin subfamily of Rab effector proteins; may be regulated by MITF; 6-50 x higher in MCs than in other tissues except duodenum (330 reads)
Taok3	270	Serine/threonine-protein kinase TAO3); Higher (3-10 x) in MCs than other tissues
Grap2	261	GRB2-related adapter protein 2; adaptor-like protein involved in leukocyte-specific protein-tyrosine kinase signaling; 50-100 x higher in MCs than in other tissues except spleen (83 reads)
Tnik	240	TRAF2 and NCK-interacting protein kinase; 4-100 x higher in MCs than in other tissues
Adcy7	233	Adenylate cyclase 7; Much higher in MCs than in other tissues; spleen closest (64 reads)
Git2	233	ARF GTPase-activating protein GIT2; 4-100 x higher in MCs than in other tissues
Syk	224	Spleen non-receptor tyrosine kinase; hematopoietic expression; Much higher in MCs than other tissue; spleen closest (55 reads)
Dennd1c	220	DENN domain-containing protein 1C; Guanine nucleotide exchange factor (GEF); Much higher in MCs than in other tissue; spleen closest (72 reads)
Vav1	204	member Dbl family of Guanine exchange factors; 2-100 x higher in MCs than in other tissues; spleen closest (78 reads)

Rin3	201	Ras and Rab interactor; Higher in MCs than in most other tissues; spleen closest (57 reads)
Arhgap25	184	Rho GTPase activating protein 25; Much higher in MCs than in other tissues except spleen (64 reads)
Casq1	173	Calsequestrin-1; controls calcium release from intracellular stores; Much higher in MCs than in most other tissues except tongue and heart (221 and 84 reads, respectively)
Fermt3	155	Fermitin family homolog 3; role in integrin signaling, cell adhesion, migration of hematopoietic cells; Higher in MCs compared to most other tissues except spleen (38 reads)
Arap3	146	Phosphoinositide binding protein; Higher in MCs than in most other tissues; uterus closest (40 reads)
Gna14	136	Guanine nucleotide-binding protein subunit alpha-14; Higher in MCs than other tissues; duodenum closest (14 reads)
Stk32b	138	Serine/threonine-protein kinase 32b; Higher in MCs than in other tissues; kidney closest (27 reads)

Suppl. Table 17. Transcript levels for genes involved in intracellular transport in mouse peritoneal MCs (from BALB/c mice). The number of normalized reads for each of the different genes is given in actual numbers (obtained from Ampliseq analyses).

Gene name	Reads	Comments
Frmd4a (	994	Role in cell polarity; Very high in MCs compared to other tissues; heart closest (35 reads)
Vat1	877	Synaptic membrane protein; High in MCs compared to other tissues; colon closest (315 reads)
Rab27b	858	Role in vesicle transport; Very high in MCs compared to other tissues except duodenum (92 reads)
Agap1	744	Role in membrane trafficking; High in MCs compared to other tissues; brain closest (184 reads)
Emilin2	708	Emilin2 (intracellular transport); Very high in MCs compared to other tissues; uterus closest (75 reads)
Coro7	617	Coronin 7; regulates Golgi and endosomal transport; High in MCs compared to other tissues; spleen closest (70 reads)
Tuba8	439	Tubulin 8; High in MCs compared to other tissues except tongue and heart (592 and 1380 reads, respectively)
Vcl	384	Vinculin; links integrin and actin; Relatively high in MCs compared to other tissues except uterus (325 reads)
Stx3	355	Syntaxin 3; role vesicular transport; Very high in MCs compared to other tissues except colon (61 reads)
Vps39	332	promotes clustering of endosomes and lysosomes; Higher (5-100 x) in MCs compared to other tissues

Mvb12b	332	Multi-vesicular body subunit 12B; High in MCs compared to other tissues except brain (291 reads)
Fam107b	341	Possible role in vesicular transport; High in MCs compared to other tissues (kidney: 75 reads)
Wipf1	237	WAS/WASL-interacting protein family member 1; cytoskeletal effects; Higher in MCs than in most other tissues (spleen: 70 reads)
Smpx	233	Small muscular protein; Much higher in MCs than in other tissues except heart and tongue (909 and 723 reads, respectively)
Nckap1l	225	Hematopoietic specific actin cytoskeletal regulator; Much higher in MCs than other tissues (spleen: 74 reads)
Syth3	218	Cytohesin protein sorting and membrane trafficking; Much higher (10-100 x) in MCs than in other tissues

Suppl. Table 18. Transcript levels for genes involved in various processes in mouse peritoneal MCs (from BALB/c mice). The number of normalized reads for each of the different genes is given in actual numbers (obtained from Ampliseq analyses).

Gene name	Reads	Comments
Lgals	2116	Galectin 1; immunosuppressive function; Higher in MCs than in most other tissues except uterus (3000 reads)
Basp1	1005	Brain acid soluble protein 1; Higher in MCs than in brain (brain; 522 reads)
Optn	774	Optineurin; unknown function; High in MCs compared to other tissues (liver: 120 reads)
Sorbs3	758	Vinexin; role in cell spreading; High in MCs compared to other tissues; uterus closest (120 reads)
Cblb	698	E3 Ubiquitin protein ligase; High in MCs compared to other tissues; uterus closest (48 reads)
Nav1	500	Neuron activator; unknown function; Very high in MCs compared to other tissues (15 x higher in MCs than brain)
Ubash3b	448	Ubiquitin associated domain; may inhibit receptor endocytosis; High in MCs compared to other tissues (brain: 53: reads)
Scn1b	358	Sodium channel beta-1; Very high in MCs compared to other tissues except brain (383 reads)
Slc31a2	353	Possible copper transporter; Very high in MCs compared to other tissues; tongue closest (31 reads)
LRMP	406	TAP independent peptide transport for MHC I; Very high in MCs compared to other tissues except spleen (100 reads).
C2	159	Complement factor C2; Much higher than other tissues except liver and uterus (50 and 54 reads, respectively)
ERV3	141	Endogenous full-length retrovirus; Much higher in MCs than in other tissues; spleen closest (0.545 normalized reads)
